# Work hours, appraisal at work, and intention to leave the medical research workforce in Japan

**DOI:** 10.1093/joccuh/uiaf044

**Published:** 2025-08-21

**Authors:** Keisuke Kuwahara, Akira Minoura, Yuhei Shimada, Yuki Kawai, Hiroko Fukushima, Makoto Kondo, Takehiro Sugiyama

**Affiliations:** Department of Epidemiology and Prevention, Center for Clinical Sciences, National Center for Global Health and Medicine, Shinjuku-ku, Japan; Department of Public Health, Yokohama City University School of Medicine, Yokohama, Japan; Department of Health Data Science, Graduate School of Data Science, Yokohama City University, Yokohama, Japan; Department of Hygiene, Public Health and Preventive Medicine, Showa University School of Medicine, Shinagawa-ku, Japan; Department of Law and Politics, The University of Tokyo, Bunkyo-ku, Japan; Diabetes and Metabolism Information Center, Research Institute, National Center for Global Health and Medicine, Shinjuku-ku, Japan; Department of Epidemiology and Prevention, Center for Clinical Sciences, National Center for Global Health and Medicine, Shinjuku-ku, Japan; Department of Pediatrics, University of Tsukuba Hospital, Tsukuba, Japan; Department of Child Health, Institute of Medicine, University of Tsukuba, Tsukuba, Japan; Department of Anatomy and Neuroscience, Graduate School of Medicine, Osaka Metropolitan University, Osaka, Japan; Diabetes and Metabolism Information Center, Research Institute, National Center for Global Health and Medicine, Shinjuku-ku, Japan; Department of Health Services Research, Institute of Medicine, University of Tsukuba, Tsukuba, Japan

**Keywords:** long working hours, performance appraisal, intention to leave, medical researchers, cross-sectional study

## Abstract

**Objectives:**

Strengthening the research workforce is essential to safeguard public health and human lives. This study examined the associations between work hours and perceived performance appraisal, and the intention to leave the medical research workforce.

**Methods:**

This cross-sectional study used data collected from medical researchers between December 2022 and January 2023. The questionnaire was distributed to participants via all 141 societies of the Japanese Association of Medical Sciences. Weekly work hours were self-reported using 10 response options. Perceived appraisal of research performance at work was assessed using 6 response options and dichotomized into inappropriately appraised (slightly disagree/totally disagree) and the rest. Intention to leave the research workforce was also self-reported and dichotomized. We calculated multivariable-adjusted odds ratios (aORs) for intention to leave, according to work hours and perceived appraisal.

**Results:**

Of 3139 participants (852 women), most (*n* = 686) worked 60-79 hours weekly. One in four (*n* = 745) felt inappropriately appraised, and 11% (*n* = 356) intended to leave. A U-shaped association was observed between work hours and intention to leave (aOR: 2.05; 95% CI, 1.12-3.73, for weekly working 100 hours or longer), although the quadratic trend was not significant (*P* = .15). The inappropriately appraised group had a 3.6 times (95% CI, 2.81-4.58) higher OR of intending to leave compared with their appropriately appraised counterparts.

**Conclusions:**

The results suggest that researchers who work long hours and feel inappropriately appraised are more likely to consider leaving the medical research workforce.

## Teaser

Key points

- Risk factors for intention to leave the medical research workforce have not been well studied.
- Although the effort-reward imbalance model has been used to explain intention to leave among medical professionals, it has not been applied to medical researchers.

- Our findings suggest that long work hours and perceived inappropriate appraisal may increase the likelihood of intending to leave the medical research workforce.

- These findings may serve as a foundation for developing strategies to retain researchers in the medical field.

## 1. Introduction

Strengthening research capacity for health has been a critical issue globally.^1^^,^^2^ This issue was particularly highlighted by the recent COVID-19 pandemic.^3^ To prevent and mitigate future pandemic crises, research is expected to play key roles.^4^ Thus, ensuring sufficient research capacity is crucial in medicine for sustainable development of the world. In general, high-income countries have greater research capacity than low- or middle-income countries.^5^^,^^6^ However, even in high-income countries, sustaining the medical research workforce is not straightforward.^7^ Therefore, it is necessary to identify modifiable factors to promote a sustainable research workforce in health and medicine.

In human resources management, intention to leave has been extensively studied among health care professionals, possibly because intention to leave can predict actual leaving at a later stage.^8^ Cause of turnover intention has been explained using the effort-reward imbalance model,^9^^,^^10^ in which workplace stress is expected to occur if efforts are not reciprocated by rewards (eg, financial rewards, such as money; rewards in social status, such as career promotion or job security; and socio-emotional rewards, such as esteem or recognition of work performance).^11^ Regarding effort, “working hard” is known to be a tip for becoming a successful researcher.^12^ In addition, the academic workforce is characterized by hypercompetiteness,^13^ indicating that a high degree of effort is required from researchers. As for rewards, a recent survey of postdoctoral researchers^14^ showed that approximately half of the respondents were not satisfied with their salary. Even professors receive poorer payments compared with doctorate-holders in other sectors (widely dispersed across industries with about one-fourth in professional, scientific, and related services, and another fourth in educational, health, and social services).^15^ Pay dissatisfaction is a known risk factor for intention to leave.^16^^,^^17^ Moreover, getting a promotion and tenure position is difficult, especially for early career researchers in academia.^18^ Taken together, esteem at work may be a more important modifiable factor affecting retention in the medical research workforce.^19^ However, no quantitative studies have examined this point in relation to leaving the research workforce. Evidence on this topic will help develop a better research system for medicine.

Here, we investigated the cross-sectional associations of work hours (as a proxy for effort) and appraisal as a researcher at work (as a reward) with the intention of leaving the medical research workforce. We used large-scale nationwide data from researchers belonging to medical societies within the Japanese Association of Medical Sciences.

## 2. Methods

### 2.1. Study design and settings

This cross-sectional study followed the Strengthening the Reporting of Observational Studies in Epidemiology (STROBE) reporting guidelines. In our analysis, we used web-based survey data obtained from medical researchers in Japan. The details of the survey procedures were explained previously.^20^ In short, the survey was performed from December 2022 to January 2023 as a part of the 31st General Assembly of the Japanese Association of Medical Sciences, a 4-yearly congress of medicine in Japan. We asked all the 141 academic societies in medicine belonging to the Japanese Association of Medical Sciences to distribute an online survey questionnaire to their members. The 141 societies, covering basic science, clinical medicine, and social medicine, are listed in the Supplementary information. According to the Japanese Medical Science Federation, the total membership of the major academic societies exceeds 1 million.

Eligible participants were informed about the present survey on the survey website and were asked to agree to participate. We excluded those who did not consent to participate in the study or whose daily work was unrelated to the research. If eligible participants took maternity or other leave, they were instructed to answer questions regarding their work before leaving. Then, we additionally excluded those who provided invalid responses, defined as the same answers to all questions. This survey was approved by the ethical committee of the National Center for Global Health and Medicine (approval number: NCGM-S-004530–01).

### 2.2. Exposures

Weekly usual work hours were self-reported using 10 response options: <30, 30 to 34, 35 to 39, 40 to 44, 45 to 49, 50 to 54, 55 to 59, 60 to 79, 80 to 99, and ≥100 hours. We indicated that “work” includes any work related to research or the job, such as self-improvement, work for an academic society, and holding seminars. We included self-improvement in work hours to avoid differential reporting of work hours as there is no consensus on the definition of physicians’ work hours, especially regarding self-improvement. The inclusion/exclusion of self-improvement, such as self-learning, conference participation, and research implementation, has been discussed.^21^ We combined the categories of <40 hours into 1 group due to the small number of participants.

**Table 1:** Characteristics of medical researchers according to their intention to leave the research workforce.

	**Intention to leave the research workforce** ^**a**^	
	**No intention**	**Some intention**
Number of participants	2783	356
Age, y		
20 to 24	2 (0.1)	0 (0)
25 to 29	40 (1.4)	8 (2.3)
30 to 34	165 (5.9)	22 (6.2)
35 to 39	347 (12.5)	62 (17.4)
40 to 44	423 (15.2)	66 (18.5)
45 to 49	428 (15.4)	51 (14.3)
50 to 54	405 (14.6)	49 (13.8)
55 to 59	383 (13.8)	34 (9.6)
60 to 64	328 (11.8)	34 (9.6)
65 to 69	147 (5.3)	16 (4.5)
70 or older	74 (2.7)	10 (2.8)
Not want to answer	41 (1.5)	4 (1.1)
Gender		
Male	1994 (71.7)	250 (70.2)
Female	751 (27.0)	101 (28.4)
Other/not want to answer	38 (1.4)	5 (1.4)
Residential area		
Three major metropolitan areas (Kanto, Kinki, Chukyo)	1617 (58.1)	179 (50.3)
Japan, others	1102 (39.6)	172 (48.3)
Overseas/not want to answer	64 (2.3)	5 (1.4)
Marital status		
Unmarried	366 (13.2)	48 (13.5)
Married	2235 (80.3)	278 (78.1)
Divorced/widowed	98 (3.5)	19 (5.3)
Other/not want to answer	84 (3.0)	11 (3.1)
Employment		
Full-time, tenured	1792 (64.4)	217 (61.0)
Full-time, fixed term	760 (27.3)	94 (26.4)
Part-time	162 (5.8)	29 (8.2)
Others	69 (2.5)	16 (4.5)
MD and/or DDS		
Yes	2011 (72.3)	316 (88.8)
No	772 (27.7)	40 (11.2)
Disciplines		
Clinical medicine	1437 (51.6)	259 (72.8)
Basic medicine	648 (23.3)	49 (13.8)
Social medicine	533 (19.2)	37 (10.4)
Others	165 (5.9)	11 (3.1)
Research effort		
≤10%	903 (32.5)	203 (57.0)
20% to 30%	898 (32.3)	81 (22.8)
40% to 60%	610 (21.9)	34 (9.6)
70% to 90%	265 (9.5)	28 (7.8)
About 100%	103 (3.7)	8 (2.3)
Not want to answer	4 (0.1)	2 (0.6)
Number of PI fundings or projects		
0	1178 (42.3)	235 (66.0)
1	740 (26.6)	73 (20.5)
2	430 (15.5)	18 (5.1)
3-4	289 (10.4)	15 (4.2)
5-9	97 (3.5)	5 (1.4)
≥10	15 (0.5)	1 (0.3)
Unclear	34 (1.2)	9 (2.5)
Number of non-PI fundings or projects		
0	999 (35.9)	177 (49.7)
1	630 (22.6)	70 (19.7)
2	407 (14.6)	37 (10.4)
3-4	443 (15.9)	38 (10.7)
5-9	170 (6.1)	17 (4.8)
10	71 (2.6)	4 (1.1)
Unclear	63 (2.3)	13 (3.7)
Job satisfaction		
Very satisfactory	403 (14.5)	39 (11.0)
Somewhat satisfactory	758 (27.2)	69 (19.4)
Not either	1130 (40.6)	125 (35.1)
Somewhat dissatisfactory	492 (17.7)	123 (34.6)
Very dissatisfactory	0 (0)	0 (0)

Abbreviations: DDS, doctor of dental surgery; MD, doctor of medicine; PI, principal investigator.

a Data are shown as *n* (%).

Perceived appraisal of research performance at work was measured using 1 question: “Do you think that you are appreciated appropriately as a researcher at work?” with 6 response options: strongly agree, moderately agree, not either, moderately disagree, strongly disagree, unclear. We reclassified the participants into 2 groups: those who answered “moderately disagree” or “strongly disagree” (inappropriately appraised) and the rest to detect clinically meaningful information. A similar questionnaire on perceived appraisal at work has been used in the effort-reward imbalance model (ie, esteem reward).^22^^,^^23^

### 2.3. Outcome

The intention to leave the research workforce was measured using the question: “Do you want to continue life as a researcher?” using the following 6 response options: strongly agree, moderately agree, not either, moderately disagree, strongly disagree, unclear. The present question for intention to leave is similar to one of the questions in the nurses’ intention to stay scale, “I will consider continuing in the current job as a nurse.”^24^ A similar question for intention to stay was also used previously.^25^ Based on the distribution of intention to stay and intention to leave (ie, strong negative correlation between them),^26^ we defined that participants who answered “moderately disagree” or “strongly disagree” to this question as having the intention to leave the research workforce.

### 2.4. Other covariates

Participants reported demographic factors including age, gender, marital status, residential area (3 major metropolitan areas [Kanto, Kinki, Chukyo], the rest of Japan, and overseas), employment status, and their medical license using the response options shown in Table 1. Medical licenses were self-reported with multiple choice; we divided participants into 2 groups: those holding degrees of MD and/or DDS (physician and/or dentist) and the rest. Participants also reported research-related factors, including research disciplines, research effort, the number of fundings and projects received as a principal investigator (PI), and the number of fundings and projects received as non-PIs. Disciplines in medicine were classified into basic medicine, clinical medicine, social medicine, and others using algorithms reported elsewhere.^20^

As a potential mediator, job satisfaction was assessed using the single item, “How satisfied are you with your current job?” with 5 response options (very satisfied, moderately satisfied, neither satisfied nor dissatisfied, moderately dissatisfied, and very dissatisfied).^27^ A single-item assessment of overall job satisfaction has been shown to be a reliable and valid measure containing different facets.^27^^,^^28^ Individuals who worked at more than 1 institution were asked to answer the question about overall job satisfaction. The response options were assigned values of 1 (very satisfied) to 5 (very dissatisfied) for easier interpretation of the results.

### 2.5. Statistical analysis

Characteristics for intention to leave the research workforce are shown as number (%). We also included participant characteristics according to exposures as shown in Supplementary Tables.

We used a multivariable-adjusted logistic regression to assess the association of work hours and performance appraisal with the intention of leaving the research workforce. The following multivariable-adjusted models were created. Model 1 was adjusted for age (continuous) and gender. In model 2, we further adjusted for residential area, marital status, employment, medical license, discipline, research effort, number of PI fundings or projects, and number of non-PI fundings or projects. *P* values for quadratic trends were calculated in the analyses of work hours, with a category of 40 to <45 hours as a reference. For individual associations of work hours and appraisal at work with intention to leave, we performed stratified analysis according to age (<40 years and 40 years or older), gender, residential area, marital status, employment status, medical license, disciplines, research effort, and number of PI fundings or projects (none and some).

Next, we examined the combined associations of long work hours (100 hours or longer per week) and appraisal at work with intention to leave to assess the influence of the effort-reward imbalance on intention to leave. The cutoff point of 100 hours was selected given the significant increase in the odds of intention to leave. The interaction by perceived appraisal at work on the association of long work hours with intention to leave was assessed in 2 ways. First, we assessed additive interaction using relative excess risk due to interaction.^29^ Then, we assessed multiplicative interaction using the interaction term of perceived workplace appraisal and work hours.

We performed a post hoc analysis to examine the mediating role of job dissatisfaction in the combined association of long working hours and appraisal at work with intention to leave. First, we examined the exposure-mediator association using linear regression and the mediator-outcome association using logistic regression in model 2, where job dissatisfaction was treated as a continuous variable. Then, we conducted a causal mediation analysis; we estimated natural direct association and natural indirect association using the Stata command “mediate”.^30^ We applied a probit model for exposure-outcome association and linear regression for exposure-mediator association with adjustment for factors in model 2.

Finally, as sensitivity analyses, we repeated the analyses for the intention to leave with work hours or perceived appraisal by changing the definitions of outcome or exposure. For the outcome, we used a more relaxed definition by reclassifying those who answered “unclear” into a group of intention to leave. For perceived appraisal, we also reclassified those who answered “unclear” into the inappropriately appraised group. *P* values <.05 (2-sided) were considered statistically significant. All analyses were performed using Stata 18.0.

## 3. Results

A flowchart of participant selection is illustrated in Figure S1. Of the 3555 respondents, we excluded 386 because they did not agree to participate in the survey or their work was unrelated to the research. Additionally, we excluded 30 respondents who provided invalid responses, resulting in 3139 participants (852 women) in the main analysis. Of these, the majority (*n* = 686) worked 60 to 79 hours per week, and 1 in 4 perceived that their research was not appraised appropriately at work (23.7%, *n* = 745). Approximately 1 in 10 respondents intended to leave the research workforce (11.3%, *n* = 356).

Participant characteristics according to intention to leave the research workforce are shown in Table 1. Researchers who intended to leave were younger and tended to live in areas other than Japan’s 3 major metropolitan areas. They were more likely to have an MD and/or DDS, and their disciplines were more likely to be clinical medicine. Their research efforts tended to be low, and they were less likely to have fundings or projects as a PI. They were also more likely to be dissatisfied with their job. The participants’ characteristics according to work hours and perceived appraisal as a researcher are shown in Tables S1 and S2, respectively.

Table 2 shows the associations of work hours and appraisal at work with the intention to leave the research workforce. Work hours showed U-shaped associations with intention to leave; after adjustment for socio-demographic factors and research-related factors, as compared with working 40 to <45 hours per week, working <40 hours per week and working ≥100 hours per week were associated with increased odds of intention to leave (adjusted odds ratio [aOR]: 1.43; 95% CI, 0.84-2.43; and aOR: 2.05; 95% CI, 1.12-3.73, respectively). However, the U-shaped associations were not statistically significant (model 2, *P* for quadratic trend = .15). Stratified analyses showed that U-shaped associations were significant for researchers living in the 3 major metropolitan areas and those with some PI fundings or projects (Table S3). When looking at the associations between appraisal at work and intention to leave, being appraised inappropriately was significantly associated with increased odds of intention to leave (aOR: 3.59; 95% CI, 2.81-4.58 in model 2) compared with their counterparts. Stratified analyses revealed similar significant results across participant characteristics (Table S4).

**Table 2:** Odds ratios (95% CIs) of intention to leave the research workforce according to work hours or workplace appraisal among medical researchers.

	***n***	**Intention to leave, *n* (%)**	**Model 1** ^**a**^	***P* value**	**Model 2** ^**b**^	***P* value**
Usual weekly work hours						
<40	244	35 (14.3)	1.61 (0.98-2.67)	.06	1.43 (0.84-2.43)	.19
40 to <45	355	35 (9.9)	1 (reference)		1 (reference)	
45 to <5h	478	54 (11.3)	1.19 (0.76-1.87)	.44	1.27 (0.79-2.02)	.32
50 to <55	481	55 (11.4)	1.21 (0.77-1.90)	.40	1.31 (0.82-2.09)	.27
55 to <60	477	56 (11.7)	1.24 (0.79-1.94)	.35	1.36 (0.85-2.17)	.20
60 to <80	686	71 (10.4)	1.06 (0.69-1.64)	.78	1.20 (0.76-1.89)	.43
80 to <100	263	26 (9.9)	1.02 (0.60-1.75)	.94	1.16 (0.66-2.03)	.61
≥100	155	24 (15.5)	1.69 (0.97-2.97)	.07	2.05 (1.12-3.73)	.019
				.07^c^		.15^c^
Appraisal as a researcher at work						
Appraised appropriately or neutrally	2394	183 (7.6)	1 (reference)		1 (reference)	
Appraised inappropriately	745	173 (23.2)	3.63 (2.89-4.56)	<.001	3.59 (2.81-4.58)	<.001

Abbreviations: DDS, doctor of dental surgery; MD, doctor of medicine; PI, principal investigator.

a Adjusted for age and gender.

b Adjusted for factors in model 1 plus residential area, marital status, employment, medical license (MD and/or DDS and the rest), discipline, research effort, number of PI fundings or projects, and number of non-PI fundings or projects.

c *P* for quadratic trend.

The combined associations of long work hours and appraisal at work with the intention to leave the research workforce are presented in Table 3. The highest OR of intention to leave was observed in a group who declared long work hours and inappropriate appraisal (aOR: 5.78; 95% CI, 2.91-11.47), followed by a group characterized by no long work hours but who were appraised inappropriately (aOR: 3.49; 95% CI, 2.72-4.49) and a group with long work hours and who were appraised appropriately or neutrally (aOR: 1.26; 95% CI, 0.61-2.61), as compared with a group with no long working hours and who were appraised appropriately or neutrally. However, neither the additive nor multiplicative interactions were statistically significant (all *P* > .05).

**Table 3:** Odds ratios (95% CIs) of intention to leave the research workforce according to combinations of long work hours and appraisal at work.

**Work hours and appraisal**	**Intention to leave, *n* (%)**	**Model 1** ^**a**^	***P* value**	**Model 2** ^**b**^	***P* value**
No long work hours, appraised^c^ (*n* = 2289)	174 (7.6)	1 (reference)		1 (reference)	
Long work hours, appraised (*n* = 105)	9 (8.6)	1.15 (0.57, 2.31)	.70	1.26 (0.61, 2.61)	.53
No long work hours, not appraised (*n* = 695)	158 (22.7)	3.56 (2.82, 4.51)	<.001	3.49 (2.72, 4.49)	<.001
Long work hours, not appraised (*n* = 50)	15 (30.0)	5.01 (2.67, 9.37)	<.001	5.78 (2.91, 11.47)	<.001
Additive interaction^d^			.43		.32
Multiplicative interaction^e^			.67		.59

Abbreviation: PI, principal investigator.

a Adjusted for age and gender.

b Further adjusted for residential area, marital status, employment, medical license, discipline, research effort, number of PI fundings and number of non-PI fundings.

c Long work hours were defined as working 100 hours or longer in a usual week; appraised was defined as appraised appropriately or neutrally.

d *P* value for additive interaction was assessed using relative excess risk due to interaction.

e *P* value for multiplicative interaction was assessed using a likelihood ratio test comparing models with and without an interaction term.

The association of a combination of long work hours and workplace appraisal with intention to leave and potential mediator is illustrated in Figure S2. Compared with a group declaring no long working hours and being appraised appropriately or neutrally, the rest experienced lesser job dissatisfaction (eg, adjusted coefficient of job dissatisfaction: −0.54 in a group with long working hours and being appraised inappropriately). Job dissatisfaction was significantly associated with increased intention to leave (aOR: 1.15). Causal mediation analysis showed that, whereas the natural direct association was significant and positive, the natural indirect association via job dissatisfaction tended to negative and significant (Table S5).

When we changed the outcome definition by reclassifying those who answered “unclear” into a group having the intention to leave, the associations of work hours and perceived appraisal with intention to leave were weakened (Table S6). In contrast, when we changed the exposure definition by reclassifying those who answered “unclear” into a group who were inappropriately appraised, the results were largely unchanged (Table S7).

## 4. Discussion

In this cross-sectional study, we found that researchers working 100 hours or more per week had significantly higher odds of intention to leave the medical research workforce. We also found that researchers who considered that their research performance was not appraised appropriately at work had significantly higher odds of intention to leave. The latter relationship was robust across diverse participant characteristics. To our knowledge, this is the first study to address the intention to leave the research workforce from the viewpoint of the effort-reward imbalance model. Of note, Japan’s research capacity has been declining,^31^ especially in medicine.^32^ The present results from Japan may help countries, including Japan, to improve their medical research workforce.

The present data showed that long work hours were associated with significantly increased odds of intention to leave among medical researchers. Existing cross-sectional studies showed that working longer hours was associated with increased intention to leave among physicians.^33^^,^^34^ Another cross-sectional study of health care employees also showed that overtime was associated with decreased odds of intention to stay.^35^ The present data fill the knowledge gap for medical researchers regarding how long working hours impact the intention to leave the job.

We also observed that short working hours (<40 hours per week) were associated with increased odds of intention to leave, although this increase was not statistically significant. Given the cross-sectional design, researchers who want to quit researcher life may have reduced their working hours, raising the possibility of reverse causation. Our results support the findings of a previous study on health care employees, which showed that a part-time job was associated with a decreased intention to stay compared with a full-time job.^35^A recent study after the COVID-19 pandemic^36^ reported that 29% of academic medicine faculty considered reducing their employment to part-time by continuing or transitioning to part-time work because of work-life balance issues. It is possible that, in our study, amid the COVID-19 pandemic, researchers working short hours might have considered leaving the research field due to work-life balance issues. Future studies should clarify the reasons for the intention to leave the research workforce.

The present results showed that researchers who considered that their research performance was not appraised appropriately at work had significantly higher odds of intention to leave the research workforce. This relationship was consistent across various characteristics of the researchers. These findings align with a prospective study among nurses showing that reward frustration at work (ie, poor salary, promotion prospects, and lack of esteem) was significantly associated with the intention to leave the nursing profession. Similarly, a cross-sectional study among workers reported that performance appraisal was associated with intention to leave.^37^ The present data extend the existing knowledge on appraisal at work and the intention to leave to medical researchers.

The present analysis for combined associations of long work hours and workplace appraisal shows that the ORs of intention to leave gradually increased from a group of no long work hours and being appraised appropriately or neutrally (reference) to a group of long work hours and being appraised inappropriately. Although neither additive nor multiplicative interactions were significant, given the changes in point estimates, our results do not deny the hypothesis that effort-reward imbalance may lead to a greater intention to leave among researchers. This finding aligns with the results of a cross-sectional study showing that effort-reward imbalance was associated with the intention to leave among nurses.^10^ Additional longitudinal studies with larger sample sizes are required to confirm this hypothesis.

In our analyses, job satisfaction somewhat mediated the association of long work hours and workplace appraisal with the intention to leave. This was simply because researchers who worked longer hours tended to be more satisfied with their jobs. Additionally, researchers who were not appropriately appraised as researchers experienced greater job satisfaction. They might attach much value to jobs other than research, such as clinical practice and education, leading to greater job satisfaction. We measured overall job satisfaction in this study rather than satisfaction with doing research. Future studies may assess research-specific satisfaction to clarify the precise mechanisms.

When we changed the outcome definition, the individual associations of work hours or appraisal were weakened. These results suggest that potential risk factors may not increase the risk of intention to leave when a more relaxed outcome definition is used. Practically, it might be important to use the original definition for intention to leave, as it directly reflects participants’ willingness to leave. In contrast, when we used the modified definition for perceived appraisal, the results did not change materially. Therefore, researchers who are unsure whether they are appropriately appraised may also be a target for intervention.

It is important to discuss potential strategies to retain researchers in the medical field, based on the identified associations. In our study, researchers working extremely long hours (≥100 hours per week) were significantly more likely to intend to leave the research profession. First, understanding the underlying reasons for their excessive work hours would provide a basis for developing countermeasures. Then, workplace policies aimed at reducing overtime work together with appropriate workload management and support in task handling^38^ would be necessary to mitigate the negative impact of long work hours. Our findings also suggest that a mismatch between researchers’ self-assessment and their performance appraisals may increase the risk of intention to leave. Regular one-on-one meetings^39^ may help address these appraisal discrepancies.

The strengths of the present study include the use of recent large-scale nationwide data from medical researchers. The survey was conducted in 141 medical associations in Japan via the Japanese Association of Medical Sciences, covering the major medical associations in Japan. However, this study has several limitations. First, the cross-sectional design cannot establish causality. As mentioned above, it is possible that researchers who intended to quit may have worked shorter hours. Second, although we constructed the present study based on the effort-reward imbalance model, we did not use validated scales to measure it. Third, the validity and reliability of the present measurement of intention to leave is unclear, although we used questionnaires for intention to leave similar to those in existing studies.^24^^,^^25^ Fourth, although self-reports of long work hours have been validated among workers in large-scale companies,^40^ the validity remains unclear in the present sample. It might be that researchers with multiple tasks, especially those dissatisfied with their work, had difficulty in accurately reporting their work hours. Fifth, unmeasured confounders (eg, annual income) may have affected the results of the present study. Sixth, the present survey was originally designed to assess perceptions of research/researcher evaluations. If researchers with negative attitudes toward the current evaluation systems were more likely to participate, then those intending to quit might have been overrepresented. Lastly, the participants were researchers from Japan. The applicability to researchers in other countries should be treated with caution.

In conclusion, the present data from medical researchers in Japan suggest that long work hours and negative appraisals at work may lead to the intention to leave the research workforce. Longitudinal studies are warranted to establish causal relationships.

## Supplementary Material

Web_Material_uiaf044

## Data Availability

The dataset used in this study can be made available to researchers upon reasonable request to Dr Takehiro Sugiyama (sugiyama.t@jihs.go.jp).
