# Supplementary material for: Work hours, appraisal at work, and intention to leave the medical research workforce in Japan
Source: J Occup Health. 2025 Aug 21;67(1):uiaf044. doi: 10.1093/joccuh/uiaf044 (PMC12397488; doi:10.1093/joccuh/uiaf044)
Supplement: Web_Material_uiaf044 [file web_material_uiaf044.zip › joh_suppl_info_20250609.docx]

Supplementary information. List of the 141 member societies of the Japanese Association of Medical Sciences (as of December 2022). Information was extracted from: https://jams.med.or.jp/en/ms.html

No.1 Japanese Society for the History of Medicine

No.2 Japanese Association of Anatomists（JAA）

No.3 The Physiological Society of Japan

No.4 The Japanese Biochemical Society（JBS）

No.5 The Japanese Pharmacological Society

No.6 The Japanese Society of Pathology

No.7 The Japanese Cancer Association

No.8 The Japanese Society of Hematology

No.9 Japanese Society for Bacteriology

No.10 The Japanese Society of Parasitology

No.11 Japanese Society of Legal Medicine

No.12 Japanese Society for Hygiene

No.13 The Japanese Society of Health and Human Ecology

No.14 Japan Society of Nutrition and Food Science

No.15 The Japanese Society of Balneology, Climatology and Physical Medicine

No.16 The Japan Endocrine Society

No.17 The Japanese Society of Internal Medicine（JSIM）

No.18 Japan Pediatric Society

No.19 The Japanese Association for Infectious Diseases

No.20 The Japanese Society for Tuberculosis and Nontuberculous Mycobacteriosis

No.21 The Japanese Society of Gastroenterology（JSGE）

No.22 The Japanese Circulation Society（JPN CIRC SOC）

No.23 The Japanese Society of Psychiatry and Neurology（JSPN）

No.24 Japan Surgical Society（JSS）

No.25 The Japanese Orthopaedic Association（JOA）

No.26 Japan Society of Obstetrics and Gynecology（JSOG）

No.27 Japanese Ophthalmological Society（JOS）

No.28 Japanese Society of Otorhinolaryngology-Head and Neck Surgery

No.29 The Japanese Dermatological Association

No.30 The Japanese Urological Association

No.31 Japanese Stomatological Society（JSS）

No.32 Japan Radiological Society（JRS）

No.33 The Association of Insurance Medicine of Japan

No.34 Japanese Society of Medical Instrumentation（JSMI）

No.35 Japanese Leprosy Association

No.36 Japanese Society of Public Health（JSPH）

No.37 The Japan Society of Medical Entomology and Zoology

No.38 Japanese Association of Transportation Medicine

No.39 The Japanese Society of Physical Fitness and Sports Medicine

No.40 Japan Society for Occupational Health

No.41 The Japan Broncho-Esophagological Society（JBES）

No.42 Japanese Society of Allergology

No.43 Japanese Society of Chemotherapy

No.44 The Japanese Society for Virology

No.45 Japanese Society of Anesthesiologists（JSA）

No.46 The Japanese Association for Thoracic Surgery（JATS）

No.47 The Japan Neurosurgical Society（JNS）

No.48 The Japan Society of Transfusion Medicine and Cell Therapy

No.49 The Japanese Society for Medical Mycology（JSMM）

No.50 The Japanese Association of Rural Medicine

No.51 The Japan Diabetes Society（JDS）

No.52 The Japanese Association of Correctional Medicine（Kyousei IG）

No.53 Japanese Society of Neurology

No.54 The Japan Geriatrics Society

No.55 The Japan Society of Human Genetics

No.56 The Japanese Association of Rehabilitation Medicine

No.57 The Japanese Respiratory Society

No.58 Japanese Society of Nephrology（JSN）

No.59 Japan College of Rheumatology（JCR）

No.60 Japanese Society for Medical and Biological Engineering（JSMBE）

No.61 The Japanese Teratology Society（JTS）

No.62 The Japan Society of Hepatology（JSH）

No.63 Japan Society of Plastic and Reconstructive Surgery

No.64 Japanese Society of Tropical Medicine

No.65 The Japanese Society of Pediatric Surgeons（JSPS）

No.66 Japanese College of Angiology（Jap. Coll. Angiol. ）

No.67 Japan Society of Perinatal and Neonatal Medicine

No.68 The Japanese Society for Artificial Organs（JSAO）

No.69 The Japanese Society for Immunology（JSI）

No.70 The Japanese Society of Gastroenterological Surgery

No.71 Japanese Society of Laboratory Medicine（JSLM）

No.72 Japanese Society of Nuclear Medicine（JSNM）

No.73 Japan Society for Reproductive Medicine（JSRM）

No.74 Japanese Association for Acute Medicine（JAAM）

No.75 Japanese Society of Psychosomatic Medicine（JSPM）

No.76 Japan Society for Healthcare Administration（JSHA）

No.77 Japan Gastroenterological Endoscopy Society（JGES）

No.78 Japan Society of Clinical Oncology（JSCO）

No.79 The Japan Society for Transplantation（JST）

No.80 Japanese Society of Occupational Medicine and Traumatology（JSOMT）

No.81 The Japanese Society for Cardiovascular Surgery（JSCVS）

No.82 The Japanese Society for Lymphoreticular Tissue Research（JSLTR）

No.83 Japan Society of Neurovegetative Research

No.84 The Japan Society of Coloproctology

No.85 The Japan Society of Ultrasonics in Medicine

No.86 Japan Atherosclerosis Society

No.87 The Japan Society for Oriental Medicine

No.88 The Japanese Society of Child Neurology

No.89 The Japanese Association for Chest Surgery

No.90 The Japan Society for Medical Education

No.91 Japan Association for Medical Informatics（JAMI）

No.92 Japan Epidemiological Association

No.93 The Japanese Society of Intensive Care Medicine

No.94 Japan Society of Smooth Muscle Research

No.95 The Japanese Society of Clinical Pharmacology and Therapeutics

No.96 The Japanese Society of Neuropathology

No.97 The Japan Stroke Society

No.98 The Japanese Society of Hypertension

No.99 The Japanese Society of Clinical Cytology

No.100 The Japanese Society for Dialysis Therapy

No.101 The Japan Society for Endoscopic Surgery

No.102 Japanese Breast Cancer Society

No.103 Japan Society for the Study of Obesity

No.104 The Japanese Society on Thrombosis and Hemostasis

No.105 The Japanese Society for Vascular Surgery

No.106 Japan Society for Laser Surgery and Medicine（JSLSM）

No.107 Japanese Society of Medical Oncology（JSMO）

No.108 The Japan Society for Respiratory Endoscopy（JSRE）

No.109 Japan Primary Care Association

No.110 Japanese Society for Surgery of the Hand

No.111 The Japanese Society for Spine Surgery and Related Research

No.112 Japanese Society for Palliative Medicine（JSPM）

No.113 Japanese Society for Radiation Oncology（JASTRO）

No.114 The Japanese Society of Clinical Sports Medicine（JSCSM）

No.115 Japanese Society for Burn Injuries

No.116 Japanese Society of Pediatric Cardiology and Cardiac Surgery

No.117 Japanese Society of Sleep Research（JSSR）

No.118 Japanese Society for Magnetic Resonance in Medicine（JSMRM）

No.119 The Japan Lung Cancer Society

No.120 Japanese Gastric Cancer Association

No.121 Japanese Society for Transplantation and Cellular Therapy（JSTCT）

No.122 Japan Society of Pain Clinicians（JSPC）

No.123 Japan Society of Metabolism and Clinical Nutrition（JSMCN）

No.124 Japan Society for Dementia Research

No.125 Japanese Association for Disaster Medicine

No.126 The Japanese Society of Pediatric Hematology/Oncology（JSPHO）

No.127 Japanese Psychogeriatric Society

No.128 Japanese Society for Parenteral and Enteral Nutrition Therapy（JSPEN）

No.129 The Japanese Society for Regenerative Medicine（JSRM）

No.130 The Japanese Society for Neuroendovascular Therapy（JSNET）

No.131 Japan Osteoporosis Society

No.132 Japanese Society for Apheresis（JSFA）

No.133 The Japan Society for Menopause and Women's Health

No.134 The Japan Epilepsy Society

No.135 Japanese Society of Interventional Radiology（JSIR）

No.136 Japan Association of Endocrine Surgery（JAES）

No.137 The Japanese Society for Bone and Mineral Research

No.138 The Japan Society of Gynecologic Oncology

No.139 Japanese Society of Hepato-Biliary-Pancreatic Surgery

No.140 Japanese Society of Clinical Neurophysiology（JSCN）

No.141 The Japan Esophageal Society
